# Public Opinion on the Sale of Crib Bumpers

**DOI:** 10.1001/jamanetworkopen.2020.8089

**Published:** 2020-06-18

**Authors:** Andrea C. Gielen, Joshua M. Sharfstein

**Affiliations:** 1 Johns Hopkins Center for Injury Research and Policy, Johns Hopkins Bloomberg School of Public Health, Baltimore, Maryland; 2 Department of Health Policy and Management, Johns Hopkins Bloomberg School of Public Health, Baltimore, Maryland

## Abstract

This survey study assesses public opinions on banning the sale of unsafe products, their attention to warning labels, and their beliefs about crib bumpers.

## Introduction

Crib bumpers have no meaningful health benefit and have been associated with the asphyxiation and suffocation deaths of more than 40 infants in the US.^1^ The American Academy of Pediatrics^2^ and Centers for Disease Control and Prevention^3^ advise against their use, and the states of Maryland, Ohio, and New York have prohibited their sale.

The US Consumer Product Safety Commission (referred to hereafter as the Commission) is the federal regulatory agency with jurisdiction over crib bumpers. In November 2016, citing safety issues, a majority of the commissioners advised against their use.^4^ However, in January 2020, the Commission held a hearing to consider adopting a manufacturing standard to increase the product’s stiffness and a warning label on proper installation.^5^ We assessed the public’s opinions on banning the sale of unsafe products, their attention to warning labels, and their beliefs about crib bumpers.

## Methods

 This survey study was approved by the Johns Hopkins University institutional review board. We added questions to an omnibus survey of adults living in the US conducted online between January 14 and 16, 2020, by The Harris Poll, which uses a large network of online panels with millions of unique respondents worldwide recruited through more than 100 different sources. All panelists completed a confirmed or double opt-in process, which is an approved proxy for informed consent that includes introductory language describing the survey. There were 3568 survey entrants, of whom 2994 qualified and 2036 completed the survey.

Single survey items assessed respondents’ use of warning labels and their opinions about the product. Respondents who reported having heard about crib bumpers were also asked 6 Likert-scale items measuring their beliefs about crib bumpers. All percentages reflect weighted data, with respondents weighted to targets from the US Census Bureau’s Current Population Survey for US adults aged 18 years and older; no statistical testing is presented. Data were analyzed using Quantum statistical software version 5.8 (Unicom). Data analysis was performed in January 2020.

## Results

Table 1 shows the demographic characteristics of the full sample of 2038 adults who completed the survey (1059 women [51.40%]) and the 176 who were parents of children younger than 2 years (107 women [54.50%]). Among all respondents, nearly one-half (969 respondents [45.80%]) were aged 18 to 44 years, and most (1174 respondents [65.30%]) had an annual household income of $50 000 or higher. Among the 176 parents, most were aged 18 to 44 years (157 respondents [86.40%]), and most (107 respondents [69.1%]) had an annual household income $50 000 or higher.

**Table 1.** Sample Characteristics^a^

Characteristic	Participants, unweighted No. (weighted %)	
	Total (N = 2038)	Parents with child aged <2 y (n = 176)
Sex		
Male	979 (48.60)	69 (45.50)
Female	1059 (51.40)	107 (54.50)
Age, y		
18-34	635 (29.60)	114 (57.60)
35-44	334 (16.20)	43 (28.80)
45-54	357 (16.50)	14 (7.40)
55-64	335 (16.80)	5 (6.20)
≥65	377 (20.90)	0
Region		
Northeast	383 (17.60)	26 (15.60)
South	870 (38.20)	89 (45.30)
Midwest	448 (21.00)	38 (22.70)
West	337 (23.20)	23 (16.40)
Annual household income, $		
<50 000	784 (30.90)	68 (30.50)
50 000-74 999	362 (16.70)	35 (20.20)
75 000-99 999	304 (13.30)	31 (15.60)
≥100 000	508 (35.30)	41 (33.30)
Education		
High school or less	572 (30.50)	57 (30.90)
Some college	676 (35.90)	58 (41.60)
College graduate or higher	790 (33.60)	61 (27.50)

^a^ To ensure representativeness, Harris Insights and Analytics uses a 2-stage approach. In stage 1, the outgoing sample is balanced demographically (on the basis of such factors as age, sex, income, race, and education) and monitored to assess sample targets while in the field. In stage 2, Harris Insights and Analytics weights the data on sample characteristics to bring them into line with their actual proportions in the population. Propensity score weighting is used to adjust for respondents’ propensity to be online. The online survey is not based on a probability sample; therefore, no estimate of theoretical sampling error can be calculated.

Among all respondents, 1372 (67.3%) said they had heard about crib bumpers and 851 (62.0%) reported ever having used them. Among parents, 133 (75.6%) said they had heard of crib bumpers, and 86 (64.7%) reported ever having used them (data not shown).

Table 2 shows that among those who had heard of crib bumpers, 966 of all respondents (70.4%) and 88 parents (66.2%) thought they were safe, 529 respondents (38.6%) and 61 parents (45.9%) thought they were dangerous, 699 respondents (51.0%) and 86 parents (64.7%) knew that infants could suffocate because of bumpers, 592 respondents (43.2%) and 56 parents (42.1%) believed that crib bumpers helped the baby sleep, 926 respondents (67.5%) and 85 parents (63.9%) believed that bumpers made the crib look better, and 809 respondents (59.0%) and 66 parents (49.6%) agreed that “people wouldn’t be able to buy crib bumpers if they were dangerous.” All respondents were asked about buying infant products, and 801 of all respondents (39.3%) and 73 parents (44.2%) said they always read all the safety information when buying infant products (Table 2). When asked whether it would be a good idea for the government to stop the selling of crib bumpers if experts determined that they have been linked to infant deaths, 1275 of all respondents (62.6%) and 113 parents (68.5%) agreed; 209 respondents (10.3%) and 23 parents (13.9%) thought it would be a bad idea.

**Table 2.** Opinions on the Sale and Safety of Crib Bumpers Among Respondents Who Had Heard of Crib Bumpers

Belief statements among those aware of crib bumpers	Participants, No. (%)^a^	
	Total (N = 1372)	Parents with child aged <2 y (n = 133)
Crib bumpers make the crib safe for babies		
Strongly or somewhat agree	966 (70.4)	88 (66.2)
Strongly or somewhat disagree	321 (23.4)	40 (30.1)
Crib bumpers make the crib look better		
Strongly or somewhat agree	926 (67.5)	85 (63.9)
Strongly or somewhat disagree	357 (26.0)	41 (30.8)
Crib bumpers can cause babies to suffocate		
Strongly or somewhat agree	699 (51.0)	86 (64.7)
Strongly or somewhat disagree	546 (39.8)	46 (34.6)
Crib bumpers help babies sleep		
Strongly or somewhat agree	592 (43.2)	56 (42.1)
Strongly or somewhat disagree	682 (49.7)	72 (54.1)
Crib bumpers make the crib dangerous for babies		
Strongly or somewhat agree	529 (38.6)	61 (45.9)
Strongly or somewhat disagree	756 (55.1)	71 (53.4)
People wouldn’t be able to buy crib bumpers if they were dangerous		
Strongly or somewhat agree	809 (59.0)	66 (49.6)
Strongly or somewhat disagree	495 (36.1)	62 (46.6)
When buying a product intended for use with an infant, how often, if at all, do you read all of the safety information on the product label?^b^		
All the time	801 (39.3)	73 (44.2)
Most of the time	429 (21.0)	55 (33.3)
Half, some, or none of the time	408 (20.0)	36 (21.8)
If experts determine that crib bumpers have been linked to the death of some infants, do you think it would be a good idea or a bad idea for the government to stop them from being sold?^b^		
Good idea	1275 (62.6)	113 (68.5)
Bad idea	209 (10.3)	23 (13.9)
Not sure	503 (24.7)	27 (16.4)

^a^ Percentages may not add to 100 because of nonresponse (decline or not applicable).

^b^ Data are for the entire cohort of 2038 respondents and 176 parents of children younger than 2 years.

## Discussion

There is consensus among pediatric and public health authorities that crib bumper pads have been linked to infant deaths and do not offer a meaningful health benefit. The findings of this national survey suggest that many parents may still purchase these products because they perceive them to increase the attractiveness of the crib, falsely perceive them to be safe, or mistakenly believe that they would have been removed from the market if they were dangerous.

The study results are limited in the extent to which we could assess parents’ actual practices of buying or using crib bumpers or other dangerous crib products, such as mesh liners, as well as the association of parents’ opinions with these practices. Nevertheless, this is the first study, to our knowledge, to assess the public’s opinion on crib bumpers, a widely used yet dangerous infant product. By an almost 5:1 margin, parents believe that if they were linked to infant deaths, crib bumpers should not be sold. Despite previously advising against crib bumper use, the Commission is considering establishing a standard far short of a ban. Our results cannot only guide public information efforts to correct misperceptions, but should also inform regulatory, legislative, and other actions on crib bumper pads, which could be undertaken by the Commission, the Juvenile Products Manufacturers Association, and product manufacturers.
